# Comparison of three serological tests for the detection of *Coxiella burnetii* specific antibodies in European wild rabbits

**DOI:** 10.1186/s12917-020-02526-w

**Published:** 2020-08-28

**Authors:** Charles Caraguel, Sarah Bassett, David González-Barrio, Peter Elsworth, Anne-Lise Chaber

**Affiliations:** 1 School of Animal and Veterinary Sciences, The University of Adelaide, Roseworthy, South Australia; 2 SALUVET, Animal Health Department, Faculty of Veterinary Sciences, Complutense University of Madrid, Ciudad Universitaria s/n, 28040 Madrid, Spain; 3 Pest Animal Research Centre, Department of Agriculture and Fisheries, Toowoomba, Queensland Australia

**Keywords:** *Coxiella burnetii*, Q fever, ELISA, Serology, European rabbit, *Oryctolagus cuniculus*

## Abstract

**Background:**

*Coxiella burnetii* causes Q fever, a zoonotic bacterial disease with a multi-host cycle and reservoirs in wild and domestic animal species. Q fever has a significant impact on the Australian public health and economy but its ecology and contributing reservoir species remain poorly understood. In Europe, rabbits (*Oryctolagus cuniculus*) were identified as a major reservoir of *C. burnetii* and it is possible that they play a similar role in Australia. In absence of commercial kit available for rabbit, the Thermo Fisher - PrioCHECK™ Ruminant Q fever Ab Plate Kit was adapted to successfully screen rabbits population in Europe. However, this assay is not accessible in Australia and we assessed the equivalency of two commercially available kits in Australia – IDEXX - CHEKIT Q Fever Antibody ELISA kit and IDVet - ID Screen® Q Fever Indirect Multi-species with the Thermo Fisher kit (reference kit).

**Results:**

A total of 94 rabbit sera were screened by all three ELISA kits using the same confirmed positive and negative controls. While the IDEXX kit failed to agree the other two assays (concordance correlation coefficient, *r*_*b*_ < 0.77), IDVet kit showed satisfactory equivalency with Thermo Fisher (*r*_*b*_ = 0.927).

**Conclusion:**

IDvet kit provides the best alternative for Thermo Fisher in the detection of *C. burnetii* specific antibodies in rabbits in Australia. Further trials are required to confirm these preliminary results due to the low seroprevalence of *Coxiella burnetii* observed in the study sera.

## Background

*Coxiella burnetii* is the cause of Q fever or coxiellosis, a zoonotic bacterial disease of public health significance. The life cycle of the *C. burnetii* include a wide range of host animal species, commonly farm livestocks, which act as reservoirs, transmitting the disease by shedding the bacterium into the environment. *C. burnetii* infections in cattle, sheep and goats are ususally asymptomatic but can cause production losses including abortions, infertility, still births, weak offsprings, metritis, mastitis and other reproductive disorders [1, 2]. *C. burnetii* can be shed in the urine, faeces, placental material and milk of infected animals [3]. The organism can persist in the environment in a spore-like form for several months increasing the likelihood of infection of a new host [4]. Heavy rainfall and high winds speeds have been thought to cause the spores to aerosolise facilitating air-bourne spread and infections through inhalation [5]. The bacterium is highly resistant to desiccation, high temperatures, disinfectants, radiation, ultraviolet light, pressure and oxidative or osmotic stress [4]. In humans, Q fever is more common in professions with close contact with livestocks or processing animal products such as farmers, abattoir workers and veterinarians [4]. In its chronic form, human Q fever may include respiratory tract infections, headaches, pyrexia, abortion, hepatitis, osteoarticular infections and endocarditis [2, 6].

In Australia, where *C. burnetii* was first isolated*,* Q fever is endemic and has a significant health and economic impact on the farming community [7]. Past studies identified macropods as a significant wildlife reservoir of *C. burnetii* in Australia with seroprevalences up to 30% [8]. Other documented reservoir hosts in Australia include bandicoots, kangaroos, possums, cats (feral and domestic) and dogs (wild and domestic) as well as arthropod vectors such as ticks [1].

In Europe, González-Barrio et al. reported European wild rabbits (*Oryctolagus cuniculus*) as a major reservoir [9]. Rabbits can develop systemic *C. burnetii* infections and females actively shed the bacterium into the environment through vaginal secretions. Rabbits are highly fecund and have unrestricted movement allowing them to spread the disease over vast areas. The European wild rabbit inhabits agricultural land throughout Australia. As such, rabbits may possilbly be another important reservoir of *C. burnetii* in Australia*.*

Because of the transient nature of *C. burnetii* infection and shedding, serology is commonly used to detect infections and prior exposure to *C. burnetii* in animal studies. However, serological kits commercially available were developed for humans and ruminants and were not intended to detect *C. burnetii* specific antibodies in rabbits. A modified version of the Enzyme-Linked Immunosorbent Assay (ELISA) Thermo Fisher - PrioCHECK™ Ruminant Q fever Ab Plate Kit has been successfully implemented in Europe to screen wild rabbit populations [9], however, this test kit is not accessible in Australia. Therefore, this study aimed at testing the equivalency of two ELISA kits commercially available in Australia, the IDEXX - CHEKIT Q Fever Antibody ELISA kit and the IDVet - ID Screen® Q Fever Indirect Multi-species to the Thermo Fisher’s kit when detecting *C. burnetii* specific antibodies in rabbits.

## Results

**Fig. 1:**
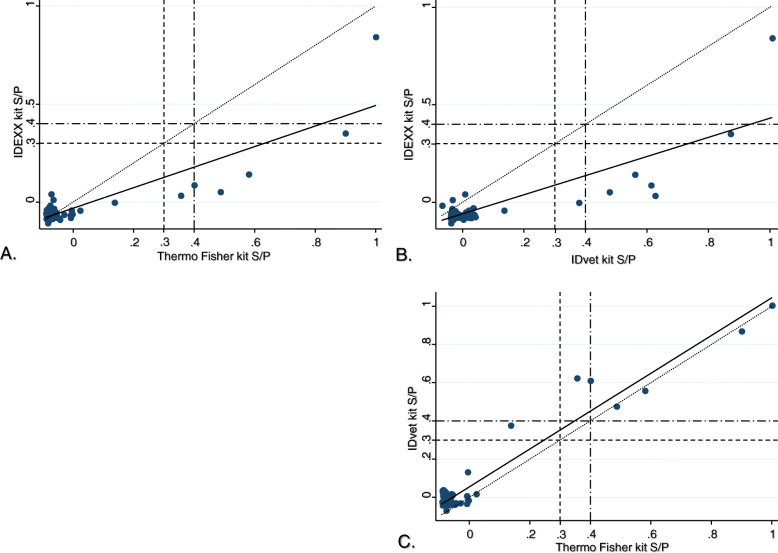
Concordance plots of the sample-to-positive ratios (S/P) between CHEKIT Q Fever Antibody ELISA kit (IDEXX kit) and PrioCHECK™ Ruminant Q fever Ab Plate Kit (Thermo Fisher kit) (**a**) or ID Screen® Q Fever Indirect Multi-species (IDVet kit) (**b**), and between IDVet and Thermo Fisher kits (**c**). The continuous line is the fitted regression line and the dotted line is the identity line. Using manufacturers’ guidelines for ruminants, illustrative S/P cutoff values were set at 0.3 (dashed line) and 0.4 (dash-dotted line) – S/*P* < 0.3: seronegative; S/*P* ≥ 0.3 and < 0.4: borderline; S/*P* ≥ 0.4: seropositive

From a study set of 94 rabbit sera, one did not yield a valid Optical Density (OD) reading for the IDEXX kit. The positive control yielded ODs of 3.129, 2.639 and 2.890, and the negative control had ODs of 0.312, 0.379 and 0.254 for Thermo Fisher, IDEXX and IDVet kit respectively. Following the same test order, sample ODs ranges (minimum-maximum) were 0.057–3.127, 0.141–2.287 and 0.052–2.907, and the sample-to-positive ratio (S/P) ranges were − 0.091-0.999, − 0.105-0.844 and − 0.067-1.006. The IDEXX S/*P* values showed moderate concordance (concordance correlation coefficient < 0.77) and a strong deviation from identity (regression slope much different from ‘1’) when compared to the others two ELISA kits (Table 1, Fig. 1a and b). The IDVet kit, however, had excellent concordance with the reference kit, Thermo Fisher, and proximated identity (regression slope = 1.02) except for a slight tendency for higher S/Ps (regression intercept = 0.056) (Table 1, Fig. 1c).

**Table 1:** Pearson’s correlarion coefficient (*r*), bias-corretion factor (*C*_*b*_), concordance correlation coefficient (*r*_*b*_ *= r x C*_*b*_*)*, reduced major axis slope and intercept estimates for each pairwise comparison of sample-to-positve ratios between CHEKIT Q Fever Antibody ELISA kit (IDEXX), ELISA kits - PrioCHECK™ Ruminant Q fever Ab Plate Kit (Thermo Fisher) and ID Screen® Q Fever Indirect Multi-species (IDvet)

Pairwise comparisons	Pearson’s correlarion coefficient (*r*)	Bias-correction factor (*C*_*b*_)	Concordance correlation coefficient (*r*_*b*_ *= r x C*_*b*_)	Regression slope	Regression intercept
IDEXX vs. Thermo Fisher	0.883	0.871	0.769	0.594	−0.030
IDvet vs. Thermo Fisher	0.969	0.957	0.927	1.021	0.056
IDEXX vs. IDvet	0.844	0.776	0.654	0.582	−0.062

## Discussion

We found that the IDVet kit provides equivalent readings to Thermo Fisher’s assay and is a satisfactory alternative to detect *C. burnetii* specific antibodies in rabbits. However, the IDEXX kit failed to reproduce the results of the reference assay and we recommend not using this kit to screen rabbits until further refinements are completed.

The use of S/P cutoff values to categorise the ELISA readings would have unlikely change these conclusions. In absence of accepted S/P cutoff values for rabbits serology, we explore a potential impact using the manufacturers’ S/P cutoffs set for ruminants - S/*P* ≥ 0.4 as seropositive, S/*P* ≥ 0.3 and < 0.4 as borderline and S/*P* < 0.3 as seronegative (Fig. 1). The IDEXX kit results would included only one seropositive serum and another one borderline, both of which were strong positive (highest S/Ps) with the Thermo Fisher and IDVet assays (Fig. 1a and b). On the other hand, the IDVet kit showed satisfactory agreement with the Thermo Fisher kit, except two samples classified as borderline by one of the two assays (Fig. 1c). Compare to the other assays, the S/Ps from the IDEXX kit were substantially weaker in both absolute (OD) and relative (S/P) terms. IDVet had slightly and systematically higher S/Ps than Thermo Fisher but this did not affect their agreement. This is potentially explained by an assumed higher affinity for pathogen specific antibody binding of the IDVet’s conjugate compared to the protein G horseradish peroxidase used in the IDEXX and Thermo Fisher assays.

The seroprevalence in the study set of sera appeared low with only seven to eight samples out of 94 (< 10%) with higher S/P (Fig. 1). Increasing the proportion of seroconverted rabbits in the testing pool would provide a better coverage of the range of antibody titres and potentially a more comprehensive estimate of the assays’ equivalency.

## Conclusions

We found that the IDVet kit provides equivalent readings to Thermo Fisher’s assay and is a satisfactory alternative to detect *C. burnetii* specific antibodies in rabbits. However, the IDEXX kit failed to reproduce the results of the reference assay and we recommend not using this kit to screen rabbits until further refinements are completed.

## Methods

### Source and testing of rabbit sera

Between 2007 and 2017, the Australian Department of Agriculture and Fisheries survey rabbit from eight locations across Queensland for other reasons than Q fever screening. Collected sera were archived and made accessible for subsequent research. A subset of 192 sera were purposively selected to cover all eight locations in absence of any Q fever history. The samples were first individually screened using PrioCHECK™ Ruminant Q fever Ab Plate Kit (Thermo Fisher, Life Technologies, Carlsbad, CA, USA) at our partner laboratory in Spain following a modified protocol for rabbit testing using a Protein G conjugate as described elsewhere [9]. Positive and negative controls were provided by the Spanish Wildlife Research Institute [9]. The positive control were sera from naturally infected rabbits confirmed with the presence of *C. burnetii* DNA by Polymerase Chain Reaction (PCR) in both spleen and vaginal swabs and the presence of antibodies against *C. burnetii* with a high Optical Density (OD) with ELISA [9]. Given the successful implementation of the Thermo Fisher kit in wild rabbit, including confirmed standards, we consider this kit as our reference assay.

Based on the Thermo Fisher test results, individual sera with OD readings > 1.0 (*n* = 6) were selected as well as a random selection of 88 of the remainig sera were selected to fit one ELISA plate and to assess the equivalence of two ELISA kits commercially available in Australia; CHEKIT Q Fever Antibody ELISA kit (IDEXX, Liebefeld-Bern, Switzerland) and ID Screen® Q Fever Indirect Multi-species (IDVet, Montpellier, France). For both tests, the same positive and negative controls as for the Thermo Fisher kit were used.

As for the Thermo Fisher kit, the IDEXX test was modified using a protein G horseradish peroxidase conjugate (Sigma-Aldrich, St Louis, MO, USA) which has a high affinity for most mammalian Immunoglobulin G (IgG) antibodies. The conjugate acts as a secondary antibody which binds to the Fragment crystallisable (Fc) region of rabbit IgG specific to *C. burnetii* present in the wells after washing*.* The plate was incubated at 37 °C following the protocol from IDEXX. The ODs were read at 450 nm using a microplate reader.

For the IDVet test, the 96 well ELISA plate was run using their multi-species horseradish peroxidase conjugate provided in the kit to trial if it had an affinity for rabbit IgG allowing it to bind. The plates were incubated at 26 °C and ODs read at 450 nm.

### Data analysis

ODs were converted into a sample-to-positive ratio (S/P) as follow:

$$ \boldsymbol{S}/\boldsymbol{P}=\frac{\boldsymbol{OD}\ \boldsymbol{sample}-\boldsymbol{OD}\ \boldsymbol{negative}\ \boldsymbol{control}}{\boldsymbol{OD}\ \boldsymbol{positive}\ \boldsymbol{control}-\boldsymbol{OD}\ \boldsymbol{negative}\ \boldsymbol{control}} $$

S/Ps between kits were compared numerically by estimating the concordance correlation coefficient (*r*_*b*_) which combined the Pearsons’s correlation coefficient (*r*) and the bias-correction factor (*C*_*b*_) (*r*_*b*_ *= r x C*_*b*_) and visually by comparing the proximity of the fitted regression line with the identity kine in the concordance correlation plots [10, 11]. All statistical analyses were performed using the statistical package Stata v15.1 (College Station, StatCorp Ltd., Texas, USA).

## Data Availability

The study datasets used and/or analysis code are available from the corresponding author upon request.

## Abbreviations

ELISA: Enzyme-Linked Immunosorbent Assay
PCR: Polymerase Chain Reaction
OD: Optical Density
S/P: Sample-to-positive ratio
Thermo Fisher: PrioCHECK™ Ruminant Q fever Ab Plate Kit
IDEXX: CHEKIT Q Fever Antibody ELISA kit
IDVet: ID Screen® Q Fever Indirect Multi-species
IgG: Immunoglobulin G
Fc: Fragment crystallisable
*r*: Pearsons’s correlation coefficient
*C*_*b*_: Bias-correction factor
*r*_*b*_: Concordance correlation coefficient
